# Medicine and Politics

**Published:** 1918-04-20

**Authors:** 


### MEDICINE AND POLITICS.

By "CADUCEUS."

A MEDICAL friend lately met after a long interval has interested me by the change in his political opinions. He used to be, from his own account and to the observation of the rest of us, a Liberal in his student days. Now he takes the *Morning Post*, hangs etchings of cathedrals in his consulting-room, thinks the House of Lords a much abler body of men than the House of Commons (for a long time thought the Grand Duke Nicholas the best general in the war), complains of the selfish wrong-doing of the working classes, and lives in dread of having his fairly considerable savings legislated away from him and his, to provide for them and theirs. Understand that the change is in no sense a deterioration. Now, equally as then, he is industrious, ascetic, parsimonious, and no snob. When I put the alteration to him, how, in the illuminating and suggestive Continental phrase, he has "moved to the Right," he protests in astonishment. "It is the world that has changed," he says, "not I."

Well, likely enough the conversatism that age with stealing steps brings to most people, is often unperceived by them. But this set me thinking how the medical profession itself has also moved away from the Left, and seemingly as unconsciously. Consider that excellent journal the *Lancet*. Would its founder, the combative reformer Wakley, recognise it now, any more than Tom Hood or Douglas Jerrold would *Punch*, whose "primness," as was admirably remarked the other day, is "excruciating"? Comparatively recently, doctors were mostly Radicals. Think of Meredith's Shrapnel. Or if that be too fine literature to be typical of the life of the mere period, take Tom Robertson's doctors—*e.g.* Dr. Brown in "Progress": or take the early novels of Mr. Weyman and Sir Anthony Hope. In these, the British doctor was not at all the congener of bankers and squires and vicars. Nowadays he—a presentable young man from Cambridge—may easily be. And still more so the lady doctor, whose father is often a man of means. Here the world as well *has* changed a little, one must grant that. Social distinctions have worn down somewhat, classes have been levelled down and up. But the forward march of medicine has made it a more prosperous profession, with the result, or concomitant, of a change in its political complexion. . . . And this, again set me wondering where do our interests lie—with the Right, or the Right Centre, or the Left Centre, or the Left? Yes, with which?

For it is not quite an easy question to answer: we shall see that when we proceed to take them one by one. Here, to begin with, is the extreme Right, the Tories, the people who used to read the old *Standard* newspaper, now defunct. These worthies feel the whole to be ever so much greater than the part. Thus, because a fleet is an old primary national need, and bacteriology a special and new-fangled affair, they would much sooner make a peer of an admiral (especially if of an old family) although a mere man of action, who never opened a book and perhaps was not even strikingly good at his job; of an ordinary admiral, I say, than of Lister. They remember, too, that intelligence is apt to run to Jacobinism. On the other hand, being authoritarians, they are for upholding all authority, including that based on the possession of special knowledge. They would support the Research Defence Society, for instance; and being sturdy vital folk, their support is worth having. Standing next to them are the Liberal Unionists. For some reason all English Universities have Unionists for their Parliamentary representatives. So far there is nothing to object to, and they likewise support research. But they wouldn't want to see more medical and fewer legal M.P.s, would they? The Liberals, now, would like that change, a change doctors think due, indeed overdue, to themselves, and in the best interests of the nation. Still, half Liberalism is Nonconformity, and Nonconformists are very loving towards quacks. So, too, are Socialists, with whom we arrive at the left flank of the political line. Have the Fabian medical men, who are reported to be standing for Labour seats at the next general election, read Mr. Shaw's recent articles in the *English Magazine*? Like Tolstoi, he hates authority, medical authority included. He would have a fashionable bonesetter given a legal qualification, *honoris causâ*. (And why, in the name of Democracy, not the thriving herbalist as well?) But then, again, we must not break with Socialists, because very plainly none but Socialistic measures can furnish anything like the funds necessary for the developments in public health work almost everyone approves of in the abstract.

Medical men have a great deal to teach each of these warring sects, I am sure of that. Medicine is the only one of the four liberal professions which is based on science, and yet, while lawyers and bishops and peers from the combatant Services are thick in the legislative chambers, there are not more than half a dozen doctors. Medicine could help to leaven Conservatism with intelligence, to knock the sentimentality out of Liberalism, to lessen the anarchism of the Socialists. Some reforms might command the support of all parties; others would have to be promoted by the help of now this one, now that. In the latter course direct Parliamentary representation is implied. With manual workers going in for direct Parliamentary representation upon a further enlarged scale, with farmers talking of it, with co-operative shoppers already fighting an election, there would be nothing anomalous in Parliamentary representatives of the medical profession. To say "nothing anomalous" is indeed putting the claim much too low. Medicine becomes more important to the national life every year; and we must take care that the interests of its exponents are safeguarded. At the time of the passing of the National Insurance Act doctors were hustled as

**MEDICINE AND POLITICS**—*(continued)*.

stevedores never could have been, just because stevedores would have had Labour M.P.s to bargain and fight for them. That very few practitioners now would like the Act altered is an accident of the particular case and makes no difference to the principle involved—which I take it is simply this; that politicians and bureaucrats must not stand to better educated men than themselves, in the relation of the armed to the defenceless. We should not then, for instance, have to man a ready-made Ministry of Health; have to sit up and beg that the heads of the medical departments of Army and Navy be admitted to the chief councils of their Services. It is worth thinking about, at least. The division lobby is a more effective place of action than the room where a Minister receives deputations.

